# Genome-wide characterization of soybean *P*_*1B*_*-ATPases* gene family provides functional implications in cadmium responses

**DOI:** 10.1186/s12864-016-2730-2

**Published:** 2016-05-20

**Authors:** Xiaolong Fang, Lei Wang, Xiaojuan Deng, Peng Wang, Qibin Ma, Hai Nian, Yingxiang Wang, Cunyi Yang

**Affiliations:** Guangdong Provincial Key Laboratory of Plant Molecular Breeding, Guangdong Sub-center of National Center for Soybean Improvement, College of Agriculture, South China Agricultural University, Guangzhou, 510642 Guangdong China; College of Life Sciences, Xinyang Normal University, Xinyang, 464000 Henan China; State Key Laboratory of Genetic Engineering and Institute of Genetics, Institute of Plant Biology, School of Life Sciences, Fudan University, Shanghai, 200438 China

**Keywords:** Soybean, HMA, P1B-ATPase, Phylogenetic analysis, Cadmium, Expression analysis

## Abstract

**Background:**

The P_1B_-ATPase subfamily is an important group involved in transporting heavy metals and has been extensively studied in model plants, such as *Arabidopsis thaliana* and *Oryza sativa*. Emerging evidence indicates that one homolog in *Glycine max* is also involved in cadmium (Cd) stress, but the gene family has not been fully investigated in soybean.

**Results:**

Here, we identified 20 heavy metal ATPase (HMA) family members in the soybean genome, presented as 10 paralogous pairs, which is significantly greater than the number in *Arabidopsis* or rice, and was likely caused by the latest whole genome duplication event in soybean. A phylogenetic analysis divided the 20 members into six groups, each having conserved or divergent gene structures and protein motif patterns. The integration of RNA-sequencing and qRT-PCR data from multiple tissues provided an overall expression pattern for the HMA family in soybean. Further comparisons of expression patterns and the single nucleotide polymorphism distribution between paralogous pairs suggested functional conservation and the divergence of *HMA* genes during soybean evolution. Finally, analyses of the *HMAs* expressed in response to Cd stress provided evidence on how plants manage Cd tolerance, at least in the two contrasting soybean genotypes examined.

**Conclusions:**

The genome-wide identification, chromosomal distribution, gene structures, and evolutionary and expression analyses of the 20 *HMA* genes in soybean provide an overall insight into their potential involvement in Cd responses. These results will facilitate further research on the *HMA* gene family, and their conserved and divergent biological functions in soybean.

**Electronic supplementary material:**

The online version of this article (doi:10.1186/s12864-016-2730-2) contains supplementary material, which is available to authorized users.

## Background

Heavy metal pollution is an increasing environmental problem affecting human health. Among the heavy metal pollutants, cadmium (Cd) has become one of the most toxic heavy metals to animals and plants owing to its high mobility and toxicity. Long-term low-level Cd exposure causes a variety of serious health risks, including emphysema, bone damage (Itai-Itai), cancer, cardiovascular disease, and irreversible chronic renal failure [1–3]. The burden of Cd on the body depends mostly on the dietary intake of plant-derived foods [3] because plants accumulate Cd in the edible parts. The roots uptake the element from Cd-contaminated soils and it is subsequently transported from roots to shoots or grains [4, 5]. Recent molecular genetic studies have identified several gene families involved in the regulation of Cd uptake or transport from *Arabidopsis*, rice, and soybean [6–8], including the P_1B_-ATPases, the natural resistance-associated macrophage proteins, and the cation diffusion facilitators [9].

P_1B_-ATPases are a subfamily of P-type ATPases and have also been described as CPx-ATPases [10], metal P-type ATPases [11], and heavy metal ATPases (HMAs) [12]. The main function of P_1B_-ATPases is involved in the transport of metal cations across biological membrane [6, 11, 13, 14]. Previous studies showed that besides conserved regions in the P-type ATPases, such as DKTGT, GDGxNDxP, PxxK, and S/TGE, P_1B_-ATPases specifically possess six to eight transmembrane segments (TMs), a HP locus, a CPx/SPC motif [15] essential for metal transport, and putative metal-binding domains in the N- and/or C-terminal regions [6, 9]. Unlike other P-type ATPase subfamilies, P_1B_-ATPases can transport multiple heavy metals, such as copper (Cu), zinc (Zn), Cd, lead (Pb), and cobalt (Co) in a wide range of organisms and are divided into two groups: Cu/Ag (Cu^+^-ATPases) and Zn/Cd/Co/Pb transporters (Zn^2+^-ATPases), based on their substrate specificity [11, 12]. In the model plants *Arabidopsis* and rice, there are eight and nine P_1B_-ATPases, respectively, which have been divided into six groups [6, 16]. Molecular genetic studies demonstrated that *Arabidopsis* HMA5, 6, 7, and 8 are specific to Cu/Ag transporters [15], while HMA2, 3, and 4 are specific to Zn/Cd transporters [17, 18]. In contrast to the other members of the *HMA* gene family, *AtHMA1* likely contributes to two types of transporter activities [19], and similar phenomena were also observed in rice HMA members [20].

Soybean is an economically and nutritionally crucial crop, and provides not only vegetable protein and edible oil, but also essential amino acids for humans and animals. Owing to the rapid urban and industrial development, Cd contamination, especially in agricultural soils, is becoming a more and more serious problem in southern China [21–23]. Soybean is a Cd-sensitive species and can accumulate Cd even at low-Cd soil concentrations. In addition, soybean genotypes are highly variable regarding Cd tolerance and grain Cd accumulation [24, 25], and several major quantitative trait loci associated with Cd responses have been identified [26, 27]. For example, *cda1* and *cd1* in chromosome 9 were associated with low-Cd concentrations in grains. After narrowing the region between the two markers, six annotated genes, including *GmHMA1/GmHMA3* (now named *as GmHMA13*), which is involved in Cd response [28, 29]. Moreover, a study showed that *GmHMA8* is able to transport Cu [30]. Thus, soybean *HMA* genes could play an important role in Cd tolerance. However, molecular features and expression information on this family are largely unknown in soybean.

Soybean is a paleopolyploid [31] with two lineage-specific whole genomic duplications (WGD), resulting in ~75 % of the total gene complement being present in multiple copies [32]. Here, we found that soybean has 20 *HMA* genes, which is more than in *Arabidopsis* or rice. For convenience, these genes were redefined from *GmHMA1* to *GmHMA20*. A phylogenetic analysis divided the 20 *GmHMA*s into six clusters. Based on the known HMA functions in *Arabidopsis* and rice, six GmHMAs (GmHMA5, 19, 13, 16, 14, and 18) were classified as Zn^2+^-ATPases, while the other HMA members in soybean were identified as Cu^+^-ATPases. Intron/exon structural patterns and conserved motifs among the 20 HMAs showed a high level of consistency between gene organization and protein structure. The expression patterns of the 20 genes in different tissues and the variation of the SNP distribution between wild and cultivated soybean lines suggest that they may be both functionally conserved and divergent. To further investigate the roles of these *HMA* genes in Cd tolerance, we examined their expression patterns in two previously identified soybean Cd-responsive genotypes, HX3 (a Cd-tolerant and low-accumulator cultivar) and ZH24 (a Cd-sensitive and high-accumulator cultivar) [33]. Analyses of the 20 *HMA*s’ expression responses to Cd stress provide an explanation for how plants deal with Cd tolerance, in terms of the HMA family, at least in the two examined contrasting soybean genotypes.

## Results

### Identification and nomenclature of *HMA* genes in soybean

To investigate the *HMA* gene family present in the soybean genome, the BLAST algorithm was used to search the phytozome website (http://www.phytozome.net/), with default settings, using the known soybean *HMA8* gene [GenBank: ABD64063.1] [30] as the query. In total, 68 genes were identified. After screening these genes for the conserved HMA motifs (DKTGT, GDGxNDxP, PxxK S/TGE, HP, and CPx/SPC), the remaining 20 genes were considered *HMA*s, which was consistent with the soybean genome annotation (GlymW82.a2.v1). A search using the BLAST algorithm, with another soybean *HMA1* [GenBank: BAL46410.1] [29] gene as the query, revealed the same *HMA* genes. To confirm that the identified proteins in soybean are potential HMAs, we performed a multiple sequence alignment of the 20 HMA protein sequences using DNAMAN and calculated manually the number of matches and mismatches based on the conserved HMA motifs, as described previously [6]. Thus, we believe that the soybean genome contains 20 putative *HMA* genes. To validate whether these genes are truly expressed, the published RNA-sequencing (RNA-seq) data from 28 developing soybean tissues were used [34]. With the exception of *GmHMA16*, which had a low expression level in the stem, the other 19 *GmHMAs* were expressed in multiple tissues (Additional file 1: Table S1).

For convenience, we redefined the soybean 20 *HMA* genes, giving them unique names from *GmHMA1* to *GmHMA20* based on their chromosomal positions, and summarized relevant characteristics of the 20 soybean HMAs (Table 1 and Additional file 1: Table S2), including annotation, chromosome location, gene loci from both Glyma1.1 and Glyma.Wm82. a2. V1 versions, protein lengths, and the features of exons and introns. To further understand the existing HMA motifs, we then performed a complete multiple protein sequence alignment of all 20 HMAs (Additional file 2: Figure S1) and identified several conserved motifs (Table 1), which were in general agreement with previously identified motifs in other HMA proteins [6]. Specifically, the 18 soybean HMAs contained all six conserved motifs, DKTGT, GDGxNDxP, PxxK, TGE, HP, and CPx/SPC, as described previously (Table 1). Interestingly, GmHMA1 lacked the PxxK motif and GmHMA4 lacked the PxxK and GDGxNDxP motifs. In addition, the replacement of DKTGT with SRQGT was also identified in GmHMA4. Thus, these proteins may have divergent functions in soybean during evolution. The DKTGT motif contains a phosphorylatable aspartate, and mutations of this aspartate inhibit the transport capabilities of AtHMA3 and AtHMA4 [35, 36]. PxxK might interact with the oxygen of the phosphate transferred from ATP [37]. D residues in GDGxNDxP were identified as binding magnesium [38]. The replacement or loss of these motifs in GmHMA1 and GmHMA4 indicates that they might lose their functions during gene duplication. Moreover, unlike other HMAs with CPx motifs, GmHMA5 and GmHMA19 possessed a SPC motif, which is a conserved characteristic of AtHMA1 and OsHMA1 [6].

**Table 1:** Characteristics of 20 GmHMAs

S.N.	Gene name	Chr. No.	Glyma1.1	Glyma.Wm82.a2.v1	Protein length	Motif					
1	GmHMA1	01	Glyma01g42790	Glyma.01G219000	913	TGE	CPC	DKTGT	HP	no	GDGINDSP
2	GmHMA2	01	Glyma01g42800	Glyma.01G219100	977	TGE	CPC	DKTGT	HP	PETK	GDGINDSP
3	GmHMA3	03	Glyma03g21650	Glyma.03G086000	954	TGE	CPC	DKTGT	HP	PVGK	GDGINDSP
4	GmHMA4	04	Glyma04g05897	Glyma.04G056100	512	TGE	CPC	SRQGT	HP	no	no
5	GmHMA5	05	Glyma05g21280	Glyma.05G098800	763	TGE	SPC	DKTGT	HP	PEDK	GEGINDAP
6	GmHMA6	05	Glyma05g24520	Glyma.05G117400	719	TGE	CPC	DKTGT	HP	PDEK	GDGINDAA
7	GmHMA7	05	Glyma05g26330	Glyma.05G132900	994	TGE	CPC	DKTGT	HP	PAGK	GDGINDSP
8	GmHMA8	06	Glyma06g05890	Glyma.06G056300	903	TGE	CPC	DKTGT	HP	PQQK	GDGINDAP
9	GmHMA9	08	Glyma08g01680	Glyma.08G013600	678	TGE	CPC	DKTGT	HP	PDQK	GDGINDSP
10	GmHMA10	08	Glyma08g07710	Glyma.08G072400	937	TGE	CPC	DKTGT	HP	PDEK	GDGINDAA
11	GmHMA11	08	Glyma08g09240	Glyma.08G087300	994	TGE	CPC	DKTGT	HP	PAGK	GDGINDSP
12	GmHMA12	09	Glyma09g05710	Glyma.09G052000	986	TGE	CPC	DKTGT	HP	PAGK	GDGINDSP
13	GmHMA13	09	Glyma09g06166	Glyma.09G055600	885	TGE	CPC	DKTGT	HP	PAEK	GDGMNDAP
14	GmHMA14	13	Glyma13g00630	Glyma.13G099600	1096	TGE	CPC	DKTGT	HP	PEDK	GDGLNDAP
15	GmHMA15	15	Glyma15g17000	Glyma.15G158300	996	TGE	CPC	DKTGT	HP	PAGK	GDGINDSP
16	GmHMA16	15	Glyma15g17375	Glyma.15G161300	793	TGE	CPC	DKTGT	HP	PSEK	GDGINDAP
17	GmHMA17	16	Glyma16g10760	Glyma.16G088300	921	TGE	CPC	DKTGT	HP	PVGK	GDGINDSP
18	GmHMA18	17	Glyma17g06800	Glyma.17G060400	809	TGE	CPC	DKTGT	HP	PEDK	GDGINDAP
19	GmHMA19	17	Glyma17g18250	Glyma.17G166800	817	TGE	SPC	DKTGT	HP	PEDK	GEGINDAP
20	GmHMA20	19	Glyma19g32190	Glyma.19G140000	984	TGE	CPC	DKTGT	HP	PDQK	GDGINDSP

Previous studies in both *Arabidopsis* and rice showed that different HMAs have distinct subcellular localizations [39]. We then predicted the subcellular localizations of the 20 HMAs using TargetP 1.1 and found that only GmHMA14 could be involved in the secretary pathway. GmHMA1, 6, 17, and 20 have a mitochondrial targeting peptide, whereas GmHMA4, 8, 10, and 19 have a chloroplast transit peptide. The localizations of the other 11 HMAs was uncertain owing to the lack of a target peptide (Additional file 1: Table S2). The subcellular localization results also support the divergent functions of HMAs under certain cellular contexts. Moreover, unlike other P-type ATPases, P_1B_-ATPases have distinct characteristics because of the reduced number of TMs, usually six to eight [15]. We used the TMHMM server to measure the number of TMs in the soybean HMAs. The result showed that, except for five members (*GmHMA4*, *5*, *8*, *16*, and *19*) in three clusters (I, II, and VI) having less than six TMs, the other members identified had six to eight TMs. In particular, GmHMA4 does not contain any TMs, suggesting that it might have lost some functions during gene expansion.

### Phylogenetic analysis and chromosomal distribution of soybean *HMA* genes

**Fig. 1:**
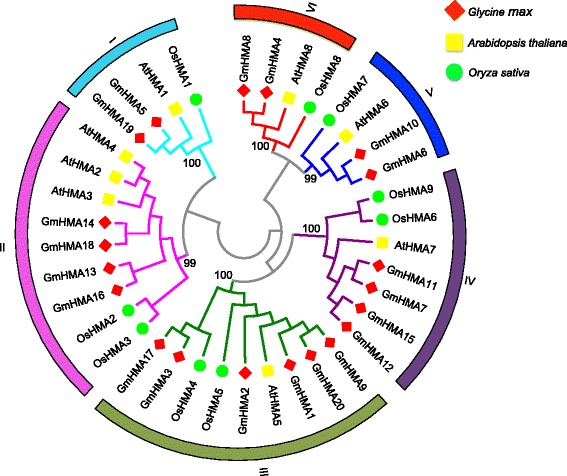
Phylogenetic analysis of *HMA* genes among *Arabidopsis*, soybean, and rice. Neighbor-joining (NJ) methods were used to construct the HMA unrooted tree using HMA amino acid sequences in soybean, *Arabidopsis*, and rice. NJ bootstrap values are presented for each main clade

To explore the evolutionary relationships of soybean *HMA* genes, we performed an unrooted phylogenetic analysis for 37 *HMA* genes from *Arabidopsis* (Additional file 1: Table S3), rice (Additional file 1: Table S4), and soybean using neighbor-joining and maximum likelihood methods (Fig. 1; Additional file 3: Figure S2). The tree topologies produced by the two methods are largely consistent with each other, with only minor differences at the interior nodes. Both trees divided the HMAs into six distinct clusters, which was consistent with previous studies [6, 20]. Each cluster included genes from *Arabidopsis*, rice, and soybean, suggesting that the same cluster had a common ancestor. Based on sequence similarities and the phylogenetic tree’s topology, the HMAs were also classified into two groups: Zn^2+^-ATPases (clusters I and II) and Cu^+^-ATPases (clusters III and VI) (Fig. 1 and Additional file 3: Figure S2). The former is responsible for Zn, Cd, Co, and Pb transport, while the latter specifically transports Cu and Ag. The relative positions in the phylogenetic tree showed that the genes in clusters III and VI were more closely related to each other than to those in cluster I or II, which is consistent with their different metal transport capabilities.

**Fig. 2:**
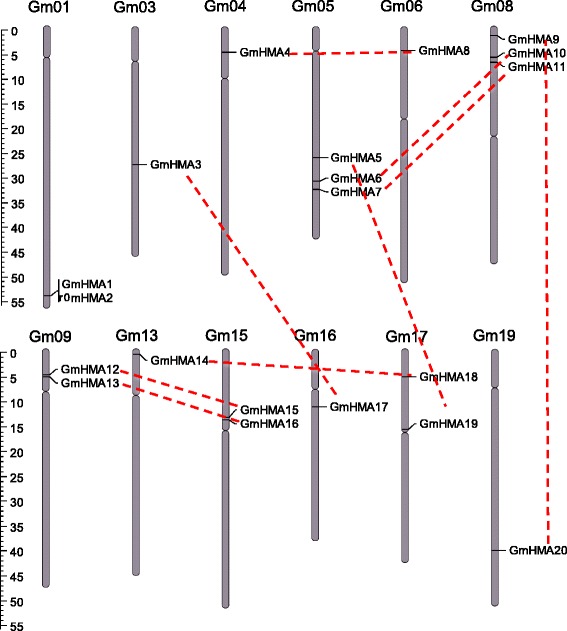
Distribution of soybean *HMA* genes on chromosomes. Chromosome size is indicated by its relative length. The scale on the left is in megabases (Mb). The vertical bars on the chromosomes indicate the positions of the centromeres. Segmental duplicated genes are indicated by red lines. The figure was produced and modified using the Map Inspector program

As mentioned earlier, soybean has more than double the number of HMA members than either *Arabidopsis* or rice, which was likely caused by the recent WGD in soybean. Gene duplication mechanisms include tandem duplication (TD) and large segmental/WGD events [40]. To examine which contributed to the expansion of the *GmHMA* gene family, a chromosome map was constructed based on the locations provided by the SoyBase (Glyma 1.1). The 20 *GmHMA* genes showed an unequal distribution along the 12 soybean chromosomes (Fig. 2). For example, chromosomes 5 and 8 had three genes, chromosomes 1, 9, 15, and 17 contained two genes, and chromosomes 3, 4, 6, 13, 16, and 19 only had single *HMA* genes (Fig. 2 and Additional file 1: Table S2). *GmHMA1* and *GmHMA2* are likely tandem-duplicated genes because both genes are linked in ~5 kb (Additional file 1: Table S2).

Recent studies indicated that ~75 % of soybean genes are paralogous pairs [41]. We detected the potential paralogous pairs among the 20 *GmHMAs* using MCScanx as described previously [42] and estimated the duplication time of paralogous pairs using KaKs_Calculator 1.2 (Additional file 1: Table S5) [43]. Subsequently, the 10 paralogous pairs and their duplication time were identified (Fig. 1 and Additional file 3: Figure S2), and included nine pairs of WGD-paralogs: *GmHMA3*/*17*, *GmHMA4*/*8*, *GmHMA5*/*19*, *GmHMA6*/*10*, *GmHMA7*/*11*, *GmHMA9/20*, *GmHMA12*/*15*, *GmHMA13*/*16*, and *GmHMA14*/*18*, and 1 TD-paralog, *GmHMA1*/*2*. By comparing the positions of the *GmHMAs* on the chromosome map (Fig. 2) and in the phylogenetic tree (Fig. 1), we found that, for *GmHMAs* that are clustered together on a chromosome, their respective paralogs were also clustered together on a different chromosome. For example, *GmHMA12* and *13* are clustered on the short arm of chromosome 9, and the corresponding paralogs *GmHMA15* and *16* are clustered together in the short arm of chromosome 15. Similarly, *GmHMA6* and *7* on chromosome 5 correspond to *GmHMA10* and *11* on chromosome 8.

### Comparisons of gene structures, protein motifs, and SNP distributions among the 20 HMAs in soybean

**Fig. 3:**
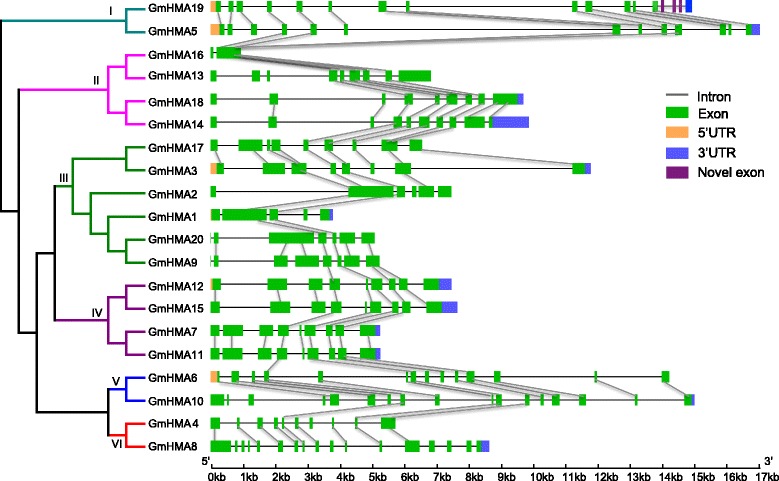
Schematic for *HMA* intron/exon structures in soybean. The gray lines indicate introns, the green boxes represent exons, the orange boxes indicate 5′ untranslated regions, the blue boxes represent 3′ untranslated regions, and the purple boxes represent novel exons. Similar exon sequences are connected by gray shaded lines

The intron/exon organization, and the intron types and numbers indicate the evolutionary history within some gene families [44–46]. We examined these features in the 20 *GmHMA* genes and observed that members of the same cluster, such as cluster 1 (*GmHMA5* and *GmHMA19*) and cluster 4 (*GmHMA12*, *GmHMA15*, *GmHMA7*, and *GmHMA11*), shared similar exon/intron structures, such as intron number and exon arrangement (Fig. 3). In contrast, some members of the same cluster showed variations in the intron/exon organization. For example, obvious changes were found in the 5′ termini of genes when compared with their paralogous pairs, such as *GmHMA17*, *GmHMA9*, and *GmHMA6. GmHMA14* was observed to have a new insertion in the last exon at the 3′ terminus, suggesting it may have obtained a different function. *GmHMA4* lacked seven exons, including those from the 2nd to 4th, and from the 14th to 17th. Additionally, *GmHMA4* had a truncation in the 1st exon compared with its paralog *GmHMA8*. (Fig. 3 and Additional file 1: Table S2). We also compared the sequence similarities of the exons among the 20 HMAs, as shown in Fig. 3. The distribution of similar exons was uneven. One gene could have only one exon, while another gene could have multiple exons. Thus, intron/exon structure likely resulted in functional conservation and/or diversification during the long-term evolution in the soybean *HMA* gene family.

**Fig. 4:**
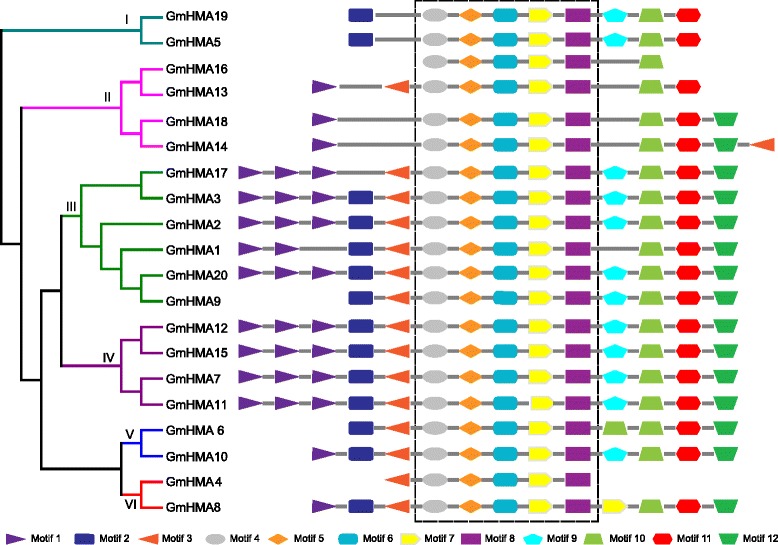
Diagrammatic representation of the 12 motif architectures of the HMA proteins in soybean. Conserved motifs in soybean HMA proteins were identified using the MEME search tool. Different motifs are indicated by different shapes with distinct colors, and the names of all of the members are shown on the left side of the figure. The motif matches are shown with a cutoff *p*-value less than 0.0001

As mentioned earlier, HMA proteins contain several highly conserved motifs, while the functions in the different clusters are obviously divergent. It is reasonable to speculate that, besides the common motifs, each group may have their own specific motifs. Therefore, a motif comparative analysis was conducted for the 20 HMAs. As shown in Fig. 4, 12 types of motifs were observed. Clearly, the 20 HMAs had the same five motifs, motifs 4–8, representing the primary domains of the P_1B_-ATPases. Among these five conserved motifs, motif 4 represents TGE, motif 6 represents CPx/SPC, motif 7 represents DKTGT, and motif 8 represents HP. However, motif 5 was not well annotated, suggesting that it could be a novel motif in the HMAs. Moreover, the same clusters had similar patterns in terms of motif types, order, and numbers (Fig. 4). For example, members of clusters I and IV shared the same motif pattern, suggesting a potential functional redundancy. However, several proteins lost or gained new motifs in either the N- or C-terminus, such as GmHMA17, 9, and 6, which lost motif 1 or 2 in their N-termini, while GmHMA14 had a new motif 3 in the C-terminus when compared with their paralogous pairs, suggesting a functional divergence between paralogous pairs. Genes with similar exon/intron structures likely have similar motif organizations. For example, genes in clusters I and IV displayed similar structural organization, and proteins in these clusters showed consistent motif arrangements.

To confirm the observed gene structure using the public dataset, we examined the 20 gene transcripts using the RNA-seq data from 28 developing soybean tissues [34]. Except for *GmHMA19*, which contained novel exons (Additional file 4: Figure S3), the exon structures of the genes were in agreement with the previous annotation.

To further investigate functional conservation and divergence among the 20 HMAs, we analyzed the distribution of SNPs previously generated from 31 wild and cultivated soybean lines [47]. In total, 864 SNPs were identified and 15 % of them were located in the exons. In addition, the SNP distribution pattern was obviously different between paralogous pairs. While *GmHMA5*/*19* and *GmHMA13*/*16* displayed similar numbers of SNPs in both exon and intron/untranslated regions, the other pairs exhibited SNP variations in either the exon or intron/untranslated regions (Additional file 1: Table S6). Specifically, both *GmHMA8* and *18* showed higher SNP numbers over the whole gene than their paralogs *GmHMA4* and *14*, respectively. A similar situation was also found between *GmHMA1* and *2* (Additional file 1: Table S6). Further analysis showed that ~55 % SNPs (72 out of 130) in exons were non-synonymous. With the exception of *GmHMA20* without non-synonymous mutation, *GmHMA3*, *4* and *18* displayed > 80 % of non-synonymous mutation rate. Thus, the different SNP distribution patterns and non-synonymous mutation rate, especially between paralogous pairs, provides additional evidence to support the functional divergence among *HMA*s during evolution.

### Expression analysis of *HMA* genes in soybean and a comparison of the corresponding homologs in *Arabidopsis* and rice

**Fig. 5:**
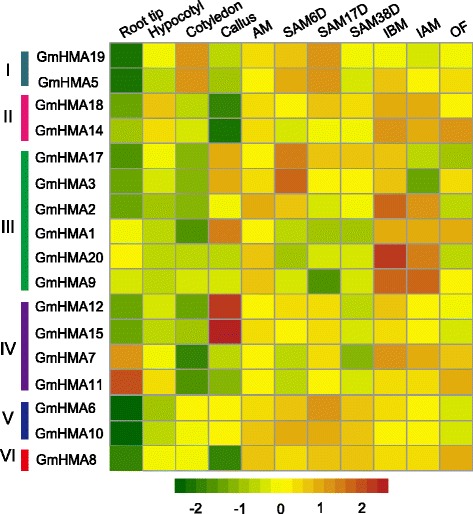
Heatmap of soybean *HMA* genes in 11 examined tissues. AM, axillary meristem; SAM6D, SAM17D, and SAM38D, shoot apical meristem at the 6-, 17-, and 38-day stages, respectively; IBM, inflorescences before meiotic stage; IAM, inflorescences after meiotic stage; OF, open flower

To further understand the potential functions of *GmHMA* genes, we investigated the expression patterns of 20 soybean *HMAs* using our previously generated RNA-seq data from 11 tissues of HX3, a soybean cultivar [41]. This dataset allowed us to detect 17 genes (Fig. 5) and, except for *GmHMA16*, *GmHMA13*, and *GmHMA4*, they showed expression in specific tissues or developmental stages as detected by RNA-seq from developing tissues of 28 soybeans (Additional file 1: Table S1). As shown in Fig. 5, genes in different clusters showed non-overlapping expression patterns, suggesting divergent functions, whereas genes in the same clusters usually displayed similar expression patterns, indicating a functional redundancy between paralogous pairs. For example, cluster IV exhibited a different expression pattern than other clusters. Furthermore, two pairs of paralogs in cluster IV were also different, but each pair showed a similar expression pattern, such as *GmHMA12* and *15* having their highest expression levels in callus, whereas *GmHMA7* and *11* had their highest levels in roots (Fig. 5).

To compare the corresponding homologs in other species, we also obtained the expression profiles of *HMA* genes in roots, stems (stem hypocotyls), leaves, flowers (inflorescences), pods, and seeds from soybean (Additional file 1: Table S7 and Additional file 5: Figure S4), *Arabidopsis* (Additional file 1: Table S8), and rice (Additional file 1: Table S9) generated by SoySeq, AtGenExpress, and PLEXdb, respectively. Most of the genes in soybean, *Arabidopsis* and rice had very broad expression spectra. Genes from the same cluster also exhibited similar expression patterns among the three species, suggesting that the genes’ functions are highly conserved among the different species. For example, most genes from clusters II–IV were preferentially expressed in the roots of the three species (Additional file 5: Figure S4 and Additional file 1: Tables S4–S6), suggesting that those genes may play an important role in root development or stress response. In addition, *GmHMA6* and *10* shared the same expression patterns in leaves, and their *Arabidopsis* counterpart, *AtHMA6*, also showed a similar pattern. However, *GmHMA19* and *5* in cluster I showed high expression levels in leaves, which was inconsistent with their counterpart *HMA1*, which was highly expressed in *Arabidopsis* flowers and in rice seeds, suggesting a functional divergence. Thus, based on the *HMAs*’ expression information among soybean, rice, and *Arabidopsis*, we speculated that the *HMA* gene family may have a distinct functional conservation and redundancy among different species.

### Validation of *GmHMA* gene expression in different tissues and an analysis of their responses to Cd stress using qRT-PCR

To validate the *GmHMA* expression information from the published database generated by SoySeq, we conducted a qRT-PCR experiment using six samples (roots, stems, leaves, flowers, pods, and seeds) from two genotypes, HX3 (a Cd-tolerant and low-accumulator cultivar) and ZH24 (a Cd-sensitive and high-accumulator cultivar) under normal conditions. Fifteen candidate genes were detected in at least one tissue (Additional file 1: Table S10). Because the public soybean data and our qRT-PCR experiment used similar tissues, the expression patterns of most of the examined genes were consistent between the two methods, except for the undetectable *GmHMA4* and *20*. Seven genes (*GmHMA1*, *2*, *7*, *9*, *11*, *12*, and *13*) and two genes (*GmHMA3* and 1*9*) were preferentially expressed in roots and leaves, respectively. These results support the reliability of our *HMA* expression information.

**Fig. 6:**
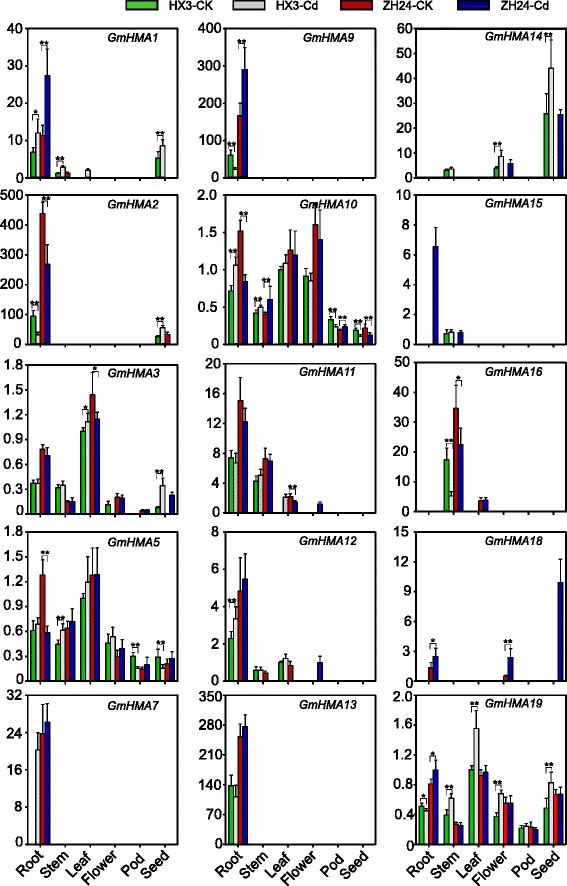
Expression patterns of *HMA* genes in six tissues in two soybean genotypes by qRT-PCR. Samples are color coded: HX3 without Cd treatment (HX3-CK) in green, HX3 with Cd treatment (HX3-Cd) in gray, ZH24 without Cd treatment (ZH24-CK) in red, ZH24 with Cd treatment (ZH24-Cd) in blue. For each gene, the expression levels obtained by normalization to ACT3 are presented on relative scales. Data are average values ± SD from three experiments, each carried out in triplicate. The significance of the changes between HX3 and ZH24 with or without Cd was assessed using the Student’s *t*-test at the level of *P* ≤ 0.05 (“*”) and *P* ≤ 0.01 (“**”)

Previous studies demonstrated that HMAs play very important roles in Cd responses in soybean [28, 29]. In this study, we compared the expression patterns of 20 soybean *HMA* genes between two contrasting genotypes, HX3 and ZH24, with and without Cd treatments. As shown in Fig. 6, the expression of 15 genes, but not *GmHMA4*, *6*, *8*, *17*, and *20*, were detected in at least one tissue. Among the detected genes, *GmHMA3*, *5*, *10*, and *19* showed wide range expression in the examined tissues, while the other 11 genes displayed tissue-preferential or -specific expression. In total, 14 genes displayed significant changes in their Cd responses between the genotypes or at least in one tissue (Fig. 6). For example, *GmHMA7*, a root-specific gene, was undetectable in HX3 without Cd treatment, but was detectable in roots treated with Cd. However, no significant expression changes were observed in ZH24 in response to Cd stress. A similar expression pattern was also found for *GmHMA12*. Interestingly, *GmHMA13* was identified as a major gene for Cd accumulation in soybean grains [28, 29] and showed root-specific expression in a Cd-unresponsive manner. The expression levels of *GmHMA1*, *2*, *3*, *14*, and *19* were significantly increased in HX3 seeds, but only *GmHMA3* and *14* were detected in ZH24, by Cd induction. Furthermore, some gene expression levels were also altered in other tissue by the Cd treatment, indicating that plant responses to Cd stress may be involved in the coordinate regulation of *HMA* gene expression. These findings provide an explanation for the molecular mechanisms of Cd responses in HX3 and ZH14.

Gene structure and protein motif organization analyses indicated that functional redundancy and divergence might occur between paralogous pairs. To test this hypothesis, a comparative analysis of expression patterns was conducted between each paralogous pair. Except for four paralogous pairs (*GmHMA3/17*, *GmHMA9/20*, *GmHMA6/10*, and *GmHMA4/8*) that had detectable expression information in at most one gene, the other paralogous pairs could be classified into three different types based on their expression change after Cd exposure. The Type I response, which displayed similar expression patterns between paralogous pairs without Cd exposure, but showed different changes after the Cd treatment, was observed for *GmHMA5/19*, *GmHMA1/2*, and *GmHMA7/11*. For example, both *GmHMA5* and *19* were expressed in six examined tissues; however, *GmHMA5* expression was significantly reduced in ZH24 roots, while *GmHMA19* displayed an obvious induction in HX3 leaves in response to Cd exposure. In addition, both *GmHMA1* and *2* were predominantly expressed in roots, but they exhibited opposite Cd responses, suggesting a functional divergence. Type II had different expression patterns with or without Cd treatments. For instance, *GmHMA13* and *16* were mainly expressed in roots and stems, respectively. However, only *GmHMA16* displayed a Cd response. A similar situation was found for *GmHMA12/15*. Type III only included *GmHMA14/18*, which showed comparative expression levels between the genotypes and the Cd treatments. Together, these results also provide an explanation of how the genetic variation in HX3 and ZH24 affected Cd responses in terms of altered paralogous gene expression levels after Cd exposure.

## Discussion

Soybean is a critical source of vegetable protein and edible oil, and has the ability to accumulate Cd in its seeds. Understanding the mechanisms of Cd accumulation in soybeans grown in Cd-contaminated soil is a very important issue for food safety. Previous studies demonstrated that P_1B_-ATPases play crucial roles in the detoxification and accumulation of Cd in several species, but mainly model plants, such as *Arabidopsis* [48] and rice [49]. Recently, extensive studies also identified *GmHMA3* [28] (*GmHMA1* [29]) and *GmHMA8* [30] from soybean, as being involved in heavy metal responses, but the molecular features and expression patterns of the *HMA* family remained to be investigated. The present study fully used molecular methods and systematic bioinformatics to analyze the HMA family in soybean as related to Cd responses.

### WGD causes the expansion of *HMA* genes in soybean

Gene duplications are considered a primary driving force in the evolution of genomes and genetic systems [50]. A recent study indicated that 90 % and 62 % of loci have undergone duplication events in *Arabidopsis* and rice, respectively [51]. Polyploidy generates massive duplications, resulting in an important source of genetic innovation [52, 53]. Soybean is a paleopolyploid [31], leading to nearly 75 % of the genes being present in multiple copies [32]. A recent study indicated that 2713 and 37,746 duplicate genes were caused by TD and WGD, respectively [41]. Here, we identified 20 members of the *HMA* family in soybean and revealed that these 20 genes could be 10 paralogous pairs. Among them, one pair is a tandem repeat, supporting the idea that TD was also involved in the expansion of the soybean *HMA* gene family. The other nine pairs evolved from segmental duplications, suggesting that segmental duplication plays a pivotal role in *HMA* gene expansion in the soybean genome. This has also been found for other gene families in soybean [44, 54].

### Possible conservation and divergence of *HMA* genes in soybean

Gene duplication is an important mechanism for understanding evolutionary novelties, while a divergence of the duplicated gene’s expression is highly correlated with a functional divergence [55, 56]. Expression patterns detected by both public RNA-seq data (generated by SoySeq) and qRT-PCR using multiple tissues provided evidence that nearly all of the soybean *HMA* genes detected had wide or distinct expression patterns, indicating that soybean *HMA* genes are likely functional after WGD events. Most soybean *HMA* genes are expressed in multiple tissues, suggesting that they have a general role in plant development. A small number of soybean *HMA* genes showed tissue-preferential expression, suggesting that those genes may have specific roles in a certain cell context.

The comparison of paralogous pairs’ expression patterns revealed that some pairs had similar expression patterns, suggesting that they might have undergone sub-functionalization, further supporting the idea that sub-functionalization might be a predominant event for WGD-duplicated genes in soybean. For example, *GmHMA5/9* are expressed in multiple tissues. Several pairs displayed different expression patterns, indicating that they might have undergone neo-functionalization after WGD in soybean. For example, *GmHAM13* was predominantly expressed in roots, while its paralog *GmHMA16* was mainly expressed in stems (Fig. 6). In addition, comparative analyses of gene expression with non-synonymous mutation rate among paralogs showed that non-synonymous mutation rate had a significant negative correlation with the gene expression levels. For instance, *GmHMA6* and *18* showed higher non-synonymous mutation rate (Additional file 1: Table S6), while displayed lower gene expression than their paralogs (*GmHMA10* and *14*) (Additional file 1: Table S7). Thus, our results provide an overall view of the functional redundancy and divergence in the HMA family that occurred after WGD in soybean.

### Potential role of *HMA* genes in soybean Cd responses

Previous studies demonstrated that GmHMA3 [28] (also known as GmHMA1 [29], now defined as GmHMA13) and GmHMA8 [30] are involved in heavy metal responses. To fully address the overall expression patterns of *HMA* in soybean, we examined their expression patterns in two genotypes with contrasting Cd responses, HX3 (Cd-tolerant cultivar) and ZH24 (Cd-sensitive cultivar) [33], with or without Cd treatments (Fig. 6 and Additional file 1: Table S10). The coordinate and distinct expression patterns of the *HMA* family members indicated that they might systematically regulate Cd redistribution. Specifically, HMA1 was identified in both *Arabidopsis* and rice, and displayed the same subcellular localization in chloroplasts [39, 57]. However, it was demonstrated to be involved in Cu and Zn transport in *Arabidopsis* and rice, respectively [39, 57]. Changes in the Cu/Zn distribution may affect Cu/Zn superoxide dismutase activities [39], finally leading to Cd resistance [33]. Here, we found, using TargetP 1.1, that the soybean homolog *GmHMA19* also had a chloroplast transit peptide (Additional file 1: Table S2). The expression pattern of *GmHMA19* resembled its *Arabidopsis* homolog, suggesting that it may also have a similar role in soybean. However, significant changes in *GmHMA19* expression under Cd treatment were found in HX3, but not in ZH24, providing an explanation for HX3’s Cd tolerance. It is likely that GmHMA19 elevates Cu/Zn superoxide dismutase activity in chloroplasts [33].

The phylogenetic tree showed that cluster II contains four soybean *HMA* genes (*GmHMA13*, *14*, *16*, and *18*) and three *Arabidopsis* genes (*AtHMA2*, *AtHMA3*, and *AtHMA4*) (Fig. 1). *GmHMA14* and *18* are homologs of *AtHMA2* and *4*, while *GmHMA13* and *16* are the *AtHMA3* homologs. In *Arabidopsis*, *AtHMA2* and *AtHMA4* have similar expression patterns, and are expressed mainly in vascular tissues [39]. Genetic studies demonstrated that both genes contribute to Cd translocation from roots to shoots. Additionally, the rice homolog *OsHMA2* also has a similar function in Cd translocation [58, 59]. In contrast, soybean *GmHMA14* was mainly expressed in the seeds of both HX3 and ZH24 (Fig. 6 and Additional file 1: Table S10). Interestingly, *GmHMA18* was only expressed in the seeds of ZH24 after Cd exposure. This finding not only indicates a functional divergence of this gene across different species, but also supports the high Cd accumulation observed in ZH24.

AtHMA3 was the first HMA identified as a Cd/Pb transporter and can rescue the *ycf1* yeast mutant strain [35]. Its expression is strictly in vascular tissues and root apices [48], which was also observed for the rice homolog of *OsHMA3* [49, 60]. A functional study demonstrated that OsHMA3 has an important role in the translocation of Cd to the tonoplast, thereby creating seeds with low Cd accumulation [60]. The soybean homolog *GmHMA16* showed a Cd-responsive expression in stems of both HX3 and ZH24, but *GmHMA13* (previously named *GmHMA1*/*3*), a soybean homolog of *AtHMA3*, did not response to Cd. In previous studies a single-base substitution was found in *GmHMA13*, a major gene controlling seed Cd accumulation, but there was no significant expression difference between the two types of soybean genotypes with contrasting Cd accumulation levels [27–30].

Moreover, the distinct expression of the *GmHMA13/16* paralogous pair suggests that they might gain new functions after duplication. *GmHMA13* was a Cd-unresponsive gene with root-specific expression, but its expression level was higher in ZH24 than in HX3, indicating that it may help ZH24 to redistribute more Cd in the roots. Whereas, *GmHMA16* is a Cd-responsive gene and its expression level in stem was also higher in ZH24 than in HX3, with or without Cd treatments, indicating that ZH24 may accumulate more Cd than HX3 in both shoots and seeds.

## Conclusions

Here, we show that the soybean genome has 20 HMA family members, presented as 10 paralogous pairs, which is significantly more than in *Arabidopsis* and rice, which is likely the result of the latest WGD in soybean. The phylogenetic analysis divided the 20 members into six groups, each having conserved or divergent gene structure and protein motif patterns. Integration of RNA-seq and qRT-PCR data from multiple tissues provided the overall expression patterns for the *HMA* family in soybean. A further comparison of the expression patterns of the paralogous pairs provides insights into the functional conservation and divergence of *HMA* genes in soybean. Finally, analyses of *HMA* expression patterns in response to Cd stress provided an explanation of how plants manage Cd tolerance, in terms of the *HMA* family, at least in the examined soybean genotypes.

## Methods

### Plant materials and growth conditions

Two soybean genotypes, HX3 (Cd tolerant cultivar) and ZH24 (Cd sensitive cultivar) were used in this study. Also, HX3 is a low grain Cd-accumulated cultivar, while ZH24 is a high grain Cd-accumulated cultivar. Seven days (d) after germination, the soybean seedlings of each cultivar were planted in two types of soils, with 0.10 ± 0.01 mg · kg^−1^ Cd and 11.32 ± 0.19 mg · kg^−1^ Cd treatments, separately. The plants were grown in greenhouse under natural condition. Roots, stems and leaves were harvested when the first trifoliate leaves were fully developed. Flowers were harvested from inflorescences. Pods and seeds were harvested when reaching 5 mm in length. All tissue samples were stored at −80 °C for RNA extraction.

### Identification and annotation of *HMA* genes in soybean genome

In order to identify the soybean *HMA* genes, we searched the entire soybean genome (Glyma1.1) by TBLASTN and BLASTP with two known soybean *HMA1/8* [GenaBank: ABD64063.1 and GenBank: BAL46410.1], at phytozome website (http://www.phytozome.net/) with default settings, separately. Alternative search using the October 2013 version (Glyma1.1) of the genome sequence was performed. Sequences were confirmed as GmHMAs using conserved sequence motifs (conserved in all P-type ATPases: DKTGT, GDGxNDxP, PxxK and S/TGE, only in P_1B_-ATPases: HP and CPx/SPC) as described previously by Williams and Mills [6].

Information of soybean *HMA* genes, including protein sequences, cDNA sequences, genomic sequences, numbers of exons and introns, first exon length and total exon length were extracted from the phytozome website (http://www.phytozome.net/). The molecular weight and isoelectric point of the protein were predicted by the ExPASy server (http://www.expasy.org/). The subcellular location predication was performed by TargetP 1.1 Server. The transmembrane segments in proteins were predicted by TMHMM server.

Exon/intron organization of soybean *HMA* genes were determined by soybean genome annotation gff file (Glyma1.1), using the online software GSDS 2.0 (http://gsds.cbi.pku.edu.cn/). For analyses of similar sequences of exons among 20 HMAs, CDS sequences of 20 *HMA* genes were extracted from the phytozome website (http://www.phytozome.net/). We used the online software MEME (http://meme-suite.org/) to identify similar sequences of exons each other (the maximum sequence length with 300 bp and the minimum sequence length with 50 bp).

Prediction of novel transcript region of the 20 *GmHMAs* were conducted using RNA-seq data extracted from the published paper [34]. These raw RNA-seq data were used to blast against the soybean genome by TopHat software [61] and viewed by inGAP software [62]. SNP information was used from the published data [47].

### Chromosome distribution and syntenic analysis of soybean *HMA* genes

Chromosomal location of the 20 soybean *HMAs* was obtained from soybean genome browse (Glyma1.1) and Map Inspect (http://www.mybiosoftware.com/mapinspect-compare-display-linkage-maps.html) was used to map the *HMA* genes onto chromosomes. We used the MCScanx software [42] to identify potential paralog pairs of *HMAs* in soybean. WGD genes and TD genes were detected with default parameters. *Ks* and *Ka*, the number of synonymous and nonsynonymous substitutions per site, were detected with KaKs_Calculator 1.2 [43]. Then, the *Ks* value was used to estimate the duplication time of duplicated *HMA* gene pairs in soybean.

### Phylogenetic tree construction of HMAs

Genome sequences of soybean *HMA* genes were retrieved from the phytozome website. The full-length amino acid sequences of rice [16], *Arabidopsis* [16] and soybean (Additional file 1: Table S11) HMAs were aligned using ClustalX [63]. The NJ tree was constructed using the Molecular Genetics Analysis (MEGA) 6.0 with the “pairwise deletion” option and “Poisson correction” model. Bootstrap support was estimated from 1000 replicates to evaluate the reliability of internal branches. The ML tree was generated using PhyML version 3.0.1 model [64].

### Expression analysis of *HMAs* genes in *Arabidopsis*, rice and soybean

To obtain expression patterns of *GmHMAs* in different tissues, we used the transcriptome data of HX3 generated from 11 meristems [41] and RNA-seq data from 28 developing soybean tissues [34]. Comparative analysis of expression profiles of homologous genes among *Arabidopsis*, rice and soybean were extracted from Soyseq (http://soybase.org/soyseq/) [65], AtGenExpress (http://jsp.weigelworld.org/AtGenExpress/resources/) [66] and PLEXdb (http://www.plexdb.org/modules/PD_browse/experiment_browser.php) database [67], respectively.

### RNA extraction and qRT-PCR

Total RNA was extracted from roots, stems, leaves, flowers, young pods and seeds of soybean plants using Trizol reagent according to the manufacturer’s instructions (Invitrogen). RNA samples were treated with RNase-free DNase I (TaKaRa) to avoid contamination of genomic DNA. The first cDNA strand was synthesized from total RNA using the MMLV-reverse transcriptase (Invitrogen) according to the protocol from the supplier. QRT-PCR analysis was carried out using the SsoFast EvaGreen Supermix kit (BIO-RAD) on a CXF96 (BIO-RAD). The specific primer sequences for 28 transcripts of 20 predicted *GmHMAs* were listed in Additional file 1: Table S12. Reaction conditions for thermal cycling were: 95 °C for 3 min, 40 cycles of 95 °C for 10 s, 57.0–61.4 °C for 10 s and 72 °C for 30 s. The annealing temperature (57.0–61.4 °C) was adjusted to suit the amplification of the individual *HMA* gene.

In real-time qRT-PCR experiment, three biological replicates within two technical replicates were performed for each sample. Expression level of the soybean *ACT3* was used as an internal control [68]. Relative expression levels of gene expression were calculated using the comparative ΔΔ*C*_*t*_ method [69]. The relative expression level (2^-ΔΔ*Ct*,HX3-CK(L)^) in the normal plant of HX3 without Cd stress was normalized to 1.

## Additional files

Additional file 1
**Table S1.** Summary of the expression values of 20 *GmHMAs* from the 28 developing soybean tissues by RNA-seq. **Table S2.** General information on the 20 *GmHMA* genes and their corresponding deduced GmHMA proteins in soybean. **Table S3.** General information on *AtHMA* genes and their corresponding deduced proteins. **Table S4.** General information on *OsHMA* genes and their corresponding deduced proteins. **Table S5.** Duplication types in the 20 *GmHMAs*. **Table S6.** Distribution of, and SNPs in, the 20 *GmHMAs* between wild and cultivated soybean lines. **Table S7.** Expression information for soybean *HMA* genes from SoySeq data. **Table S8.** Expression information for *Arabidopsis HMA* genes from AtGenExpress data. **Table S9.** Expression information for rice *HMA* genes from PLExdb data. **Table S10.** Expression information for soybean *HMA* genes by qRT-PCR. **Table S11.** Protein sequences of the 20 GmHMAs. **Table S12.** Primers used for qRT-PCR. (XLSX 63 kb) Additional file 2: Figure S1. Complete multiple sequence alignment of the HMA domains of the 20 proteins by DNAMAN. The sequence alignment was conducted by DNAMAN with the default settings. The similarity of sequences was showed by different background colors: dark blue > pink > light green. (EMF 2587 kb) Additional file 3: Figure S2. Maximum likelihood tree of *HMA* genes among soybean, *Arabidopsis*, and rice. MJ methods were used to construct the *HMA* unrooted tree using HMA amino acid sequences from soybean, *Arabidopsis*, and rice. (PDF 343 kb) Additional file 4: Figure S3. Read alignment reveals new transcriptional regions. The structure of Glyma.17G166800 (*GmHMA19*) displayed by inGAP software. The red and blue bars represent short reads. Novel exons are indicated by the red dashed boxes. (PDF 14 kb) Additional file 5: Figure S4. Heat map of *HMA* genes in five soybean tissues using data from SoySeq. The I–VI clades were divided based on the phylogenetic analysis in Fig. 1. The color gradient represents the log2-transformed RPKM values. (PDF 370 kb)

## Abbreviations

CDFs: cation diffusion facilitators
Cu/Zn-SOD: Cu/Zn superoxide dismutase
HX3: Huaxia3
ML: maximum likelihood
NJ: neighbor-joining
Nramps: natural resistance-associated macrophage proteins
TMs: transmembrane segments
ZH24: Zhonghuang24
