# Supplementary figures and images for: Genome-wide characterization of soybean P1B-ATPases gene family provides functional implications in cadmium responses
Source: BMC Genomics. 2016 May 20;17:376. doi: 10.1186/s12864-016-2730-2 (PMC4874001; doi:10.1186/s12864-016-2730-2)

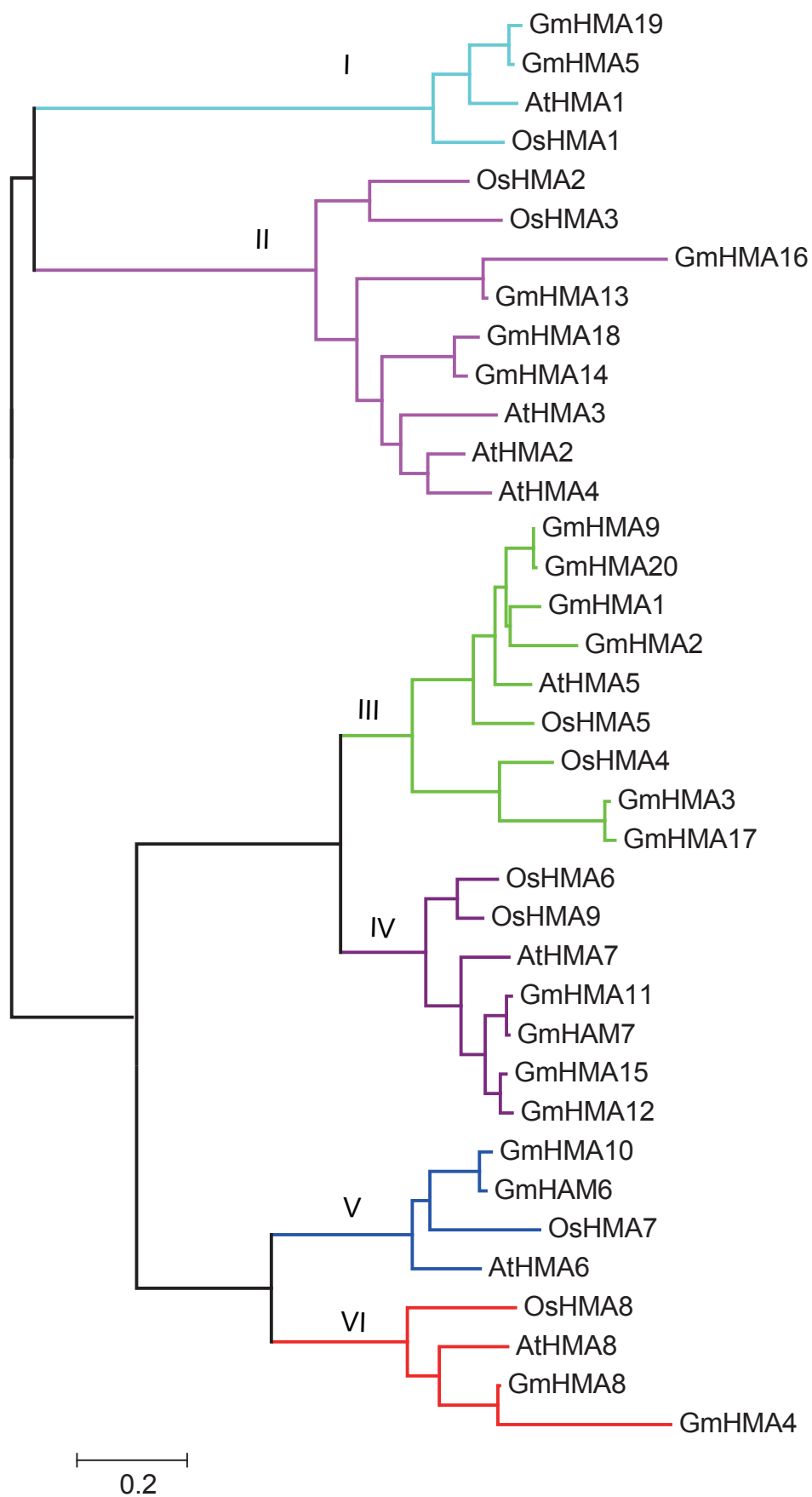

Supplement: Additional file 3: Figure S2. — Maximum likelihood tree of HMA genes among soybean, Arabidopsis, and rice. MJ methods were used to construct the HMA unrooted tree using HMA amino acid sequences from soybean, Arabidopsis, and rice. (PDF 343 kb) [file 12864_2016_2730_MOESM3_ESM.pdf]

Glyma.17G166800

Sample: flower2

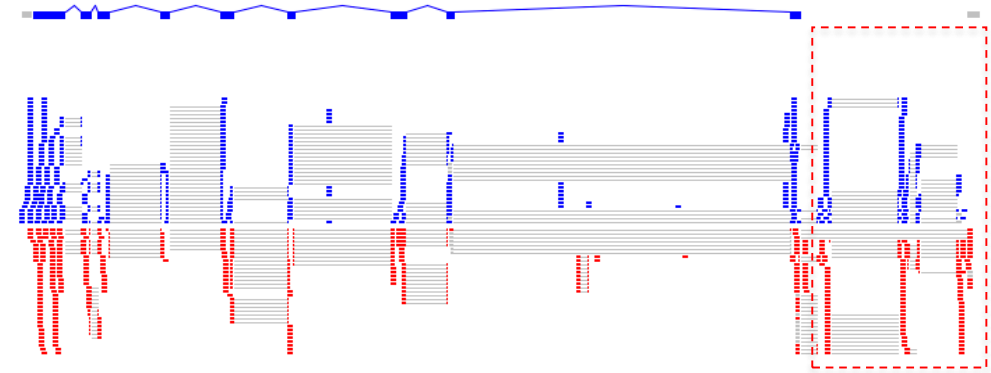

New transcription region

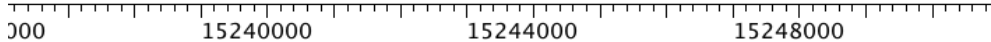

Supplement: Additional file 4: Figure S3. — Read alignment reveals new transcriptional regions. The structure of Glyma.17G166800 (GmHMA19) displayed by inGAP software. The red and blue bars represent short reads. Novel exons are indicated by the red dashed boxes. (PDF 14 kb) [file 12864_2016_2730_MOESM4_ESM.pdf]

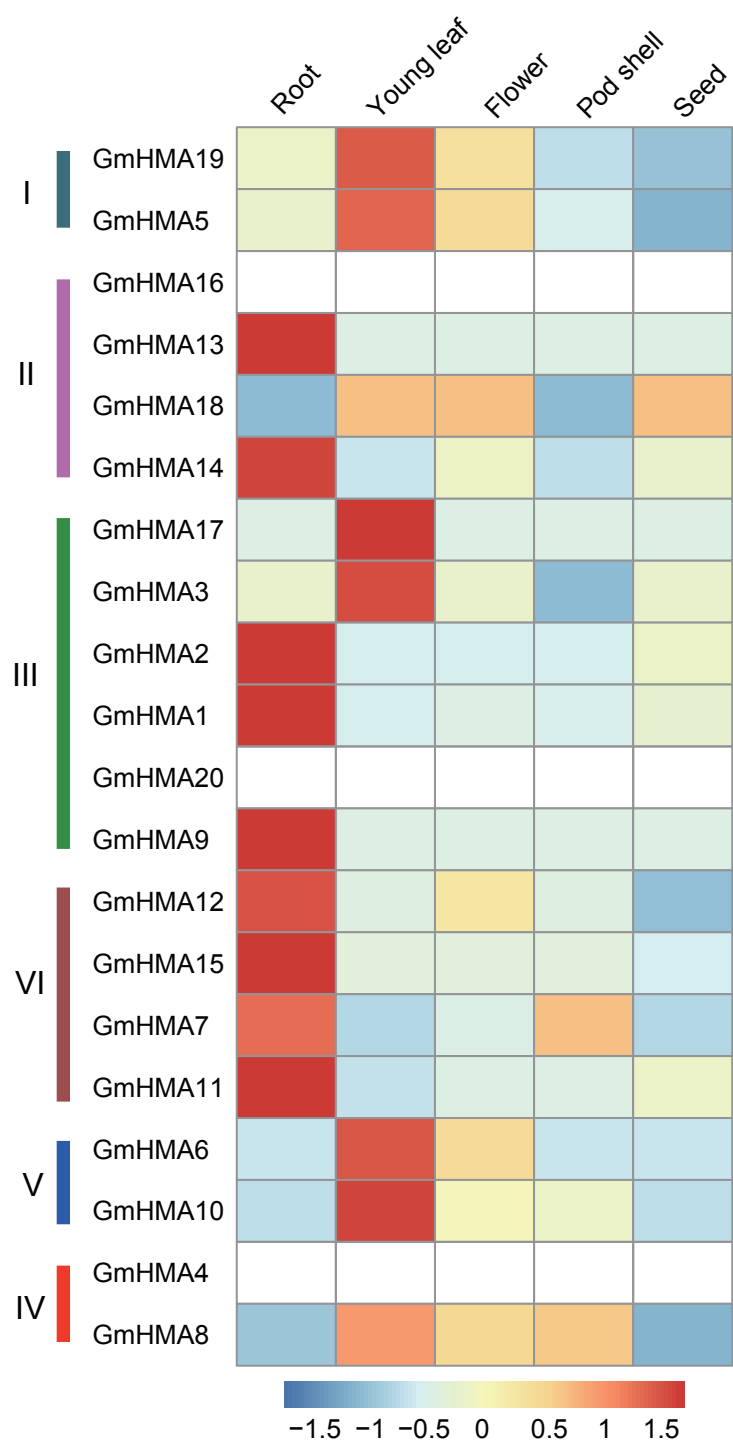

Supplement: Additional file 5: Figure S4. — Heat map of HMA genes in five soybean tissues using data from SoySeq. The I–VI clades were divided based on the phylogenetic analysis in Fig. 1. The color gradient represents the log2-transformed RPKM values. (PDF 370 kb) [file 12864_2016_2730_MOESM5_ESM.pdf]
